# Prostaglandin E2 and Cancer: Insight into Tumor Progression and Immunity

**DOI:** 10.3390/biology9120434

**Published:** 2020-12-01

**Authors:** Federica Finetti, Cristina Travelli, Jasmine Ercoli, Giorgia Colombo, Erica Buoso, Lorenza Trabalzini

**Affiliations:** 1 Department of Biotechnology, Chemistry and Pharmacy, University of Siena, 53100 Siena, Italy; ercoli3@student.unisi.it; 2 Department of Pharmaceutical Sciences, University of Pavia, 27100 Pavia, Italy; cristina.travelli@unipv.it (C.T.); erica.buoso@unipv.it (E.B.); 3 Department of Pharmaceutical Sciences, University of Piemonte Orientale, 28100 Novara, Italy; giorgia.colombo@uniupo.it

**Keywords:** prostaglandin E2, tumor inflammation, angiogenesis, metastasis, EP receptor, tumor microenvironment, cancer-related inflammation, immunosuppression

## Abstract

**Simple Summary:**

Inflammation is assessed as a hallmark of cancer and it is now widely recognized that there exists a direct causal link between inflammation and tumors. Among the inflammatory mediators, prostaglandin E2 (PGE2), the major product of cyclooxygenases (COXs), plays a pivotal role in tumor progression. Numerous pieces of evidence suggest that drugs, such as aspirin and non-steroidal anti-inflammatory drugs (NSAIDs) that inhibit PGE2 production, may exert a protective effect against tumor initiation and may play a role during tumor progression. In fact, a number of studies suggest that PGE2 increases tumor growth and invasion, reduces apoptosis, increases metastasis and angiogenesis, and suppresses antitumor immunity. In this review, we describe the current knowledge on the pro-tumoral activity of PGE2 focusing on its role in cancer progression and in the regulation of the tumor microenvironment.

**Abstract:**

The involvement of inflammation in cancer progression has been the subject of research for many years. Inflammatory milieu and immune response are associated with cancer progression and recurrence. In different types of tumors, growth and metastatic phenotype characterized by the epithelial mesenchymal transition (EMT) process, stemness, and angiogenesis, are increasingly associated with intrinsic or extrinsic inflammation. Among the inflammatory mediators, prostaglandin E2 (PGE2) supports epithelial tumor aggressiveness by several mechanisms, including growth promotion, escape from apoptosis, transactivation of tyrosine kinase growth factor receptors, and induction of angiogenesis. Moreover, PGE2 is an important player in the tumor microenvironment, where it suppresses antitumor immunity and regulates tumor immune evasion, leading to increased tumoral progression. In this review, we describe the current knowledge on the pro-tumoral activity of PGE2 focusing on its role in cancer progression and in the regulation of the tumor microenvironment.

## 1. Introduction

The involvement of inflammation in cancer progression was first described in 1863 by Rudolf Virchow. He observed that infiltrated immune cells reflect the place where cancer lesions appear in the inflamed tissue and hypothesized that chronic inflammation is a condition that predisposes one to cancer development. Most recent observations revealed that there is a direct causal link between inflammation and cancer: it is estimated that primary infections (such as *Helicobacter pylori*, hepatitis B and C viruses) and inflammatory responses are linked to 7% to 30% of cancer deaths worldwide [1,2]. Consistently, epidemiological observation showed that daily aspirin or other non-steroidal anti-inflammatory drugs (NSAIDs) reduced deaths due to several common cancers [3,4,5,6,7], indicating that cyclooxygenase (COX) inhibition and the reduction of its main metabolic product, prostaglandin E2 (PGE2), may prevent solid-organ cancers. 

## 2. Prostaglandin E2 Biosynthesis and Functions

Several biological activities have been attributed to PGE2 both in physiological and pathological conditions. In physiological processes, PGE2 regulates fever, kidney function, pain, mucosal integrity, blood vessel homeostasis, and inflammation. In pathological conditions, as in cancer, PGE2 is produced by cancerous stromal cells and enhances tumor cell proliferation and survival, promotes angiogenesis, and induces metastasis. During tumor progression, PGE2 exerts its activity through ligation with four E-type prostanoid (EP) receptors 1–4 (EP 1–4), by acting on releasing cells (autocrine mechanism) and neighboring cells (paracrine mechanism) [8]. 

PGE2 belongs to the prostanoid family of lipids, a subclass of eicosanoids produced by oxidation of 20-carbon essential fatty acids that are localized within cell membranes. Prostanoids are synthesized by sequential actions of different and highly specific enzymes. Their synthesis is initiated after the release of arachidonic acid (AA) from membrane lipids by phospholipases A2 (PLA2) family members. 

**Figure 1:**
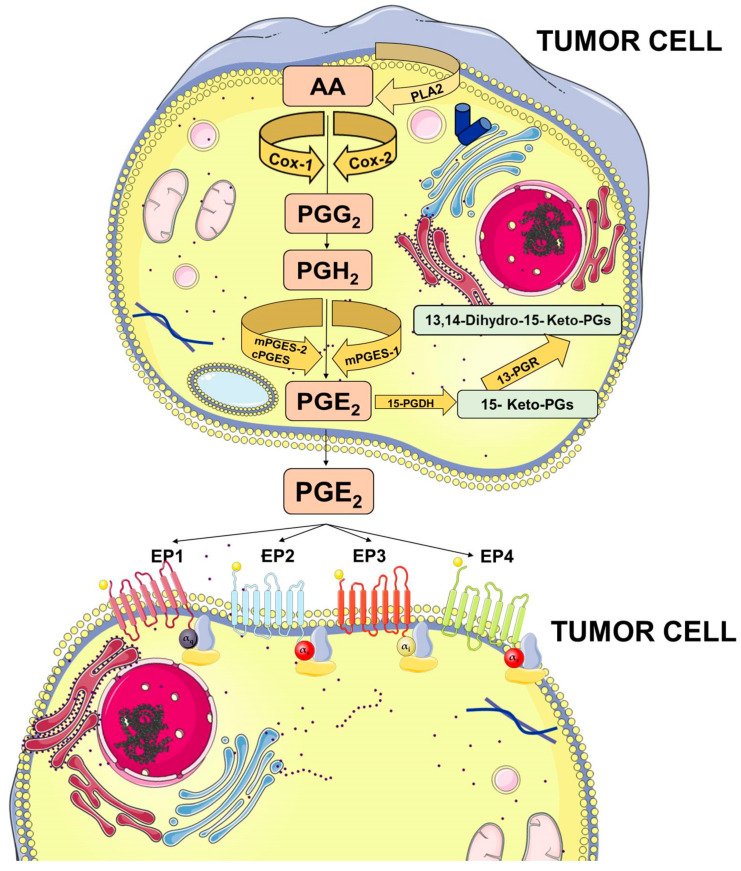
Prostaglandin E2 (PGE2) biosynthesis. Arachidonic acid (AA) is a polyunsaturated fatty acid that constitutes the phospholipid domain of most cell membranes and is released from the cellular membranes by cytoplasmic phospholipases A2 (PLA2). Free AA can be metabolized to PGE2 through the cyclooxygenase (COX) pathway. In this pathway, the key step is the enzymatic conversion of AA to the intermediate prostaglandin G2 (PGG2), which is then reduced to the intermediate PGH2 by the peroxidase activity of COX. PGH2 is sequentially metabolized to PGE2 by specific PGE synthases (cytosolic PGE synthase (cPGES), microsomal PGE synthase-1 (mPGES1), and mPGES2). PGE2 exerts its effects through ligation with four G-protein-coupled receptors (GPCRs), EP1–EP4. Each E-type prostanoid (EP) receptor couples to distinct signaling pathways. This figure was created using Servier Medical Art templates, which are licensed under a Creative Commons Attribution 3.0 Unported License; https://smart.servier.com.

Membrane-released AA is rapidly oxidized into the unstable metabolite, prostaglandin G2 (PGG2), which is subsequently reduced to PGH2. Both steps are sequentially catalyzed by the COX enzymes. COX-1 is constitutively expressed at basal levels in many cells, generating low levels of PGs that are cytoprotective and maintain body homeostasis. In contrast, COX-2 is normally absent in most cells and it is induced in response to a variety of stimuli including growth factors and cytokines [9,10,11]. Once synthesized, PGH2 is rapidly converted into PGE2 by three distinct terminal synthases (Figure 1). 

These synthases include microsomal PGE synthase-1 (mPGES-1), mPGES-2, and cytosolic PGE synthase (cPGES) and are tightly regulated under various conditions. mPGES-1 is frequently induced concomitantly with COX-2 by several proinflammatory stimuli to generate a transient increase of PGE2 levels [12,13]. The levels of PGE2 can also be regulated by its metabolic turnover. The activation of two key catabolic enzymes, 15-hydroxyprostaglandin dehydrogenase (15-PGDH) and 15-ketoprostaglandin-13-reductase (13-PGR), can essentially eliminate the biological activity of PGE2 [14]. 

Following synthesis, the physiological activity of PGE2 is mediated by the activation of downstream signaling cascades via seven transmembrane G-protein coupled receptors (GPCRs), referred as the EP receptors. The EP receptor family consists of four isoforms (EP1-4) coupled to heterotrimeric G proteins containing stimulatory (GαS) or inhibitory (Gαi) subunits that can modulate the levels of Ca2+, cyclic AMP (cAMP), and inositol phosphate, thus, activating divergent downstream signaling pathways [15] (Figure 1). The interaction between PGE2 and EP receptors is dependent on cell and tissue type and location. On cancer cells, the expression and localization of EP receptors may be variable and may influence cell response to PGE2. Specific effects depending on the activation of the different EP receptor subtype have been described, including EP1-dependent tumor cell migration and invasion, EP2-induced angiogenesis and suppression of the anti-tumor immune response, and, finally, EP4-related tumor cell migration and metastasis. The role of the EP3 receptor has yet to be clarified [15].

## 3. Prostaglandin E2 and Cancer

PGE2 is the most abundant prostaglandin that has been found in various human malignancies. Inhibition of its production by unselective COX inhibitors such as aspirin or other NSAIDs have been associated with a reduced risk of colon, breast, lung, prostate, and other solid cancers and their recurrence (see Table 1) [16,17,18]. Furthermore, it has been reported that COX-2 and/or mPGES-1 are constitutively expressed in several cancers, including non-small cell lung cancer [19,20], colorectal cancer [21,22], breast cancer [23,24], prostate cancer [25,26], melanoma [27], and hepatocellular carcinoma [28,29], suggesting that the COX-2/mPGES-1/PGE2 pathway is linked to the neoplastic progression. To outline the importance of this pathway, several efforts have been conducted to develop selective COX-2 inhibitors lacking the side effects of unselective COX inhibitors and provided with specific activities [30]. Among these, celecoxib showed antitumoral activity (see Table 1), being able to reduce the risk of colon, breast, prostate, and lung cancer [31]. However, long-term consumption of COX-2 inhibitors presents important side effects [30] and their use as anticancer agents has to be better investigated.

Several animal models have been developed and used to assess the efficacy of COX inhibition in vivo by using low-dose aspirin or NSAIDs, and to elucidate the molecular mechanisms of PGE2-induced tumor progression. 

A large number of studies have been conducted to reveal the role of PGE2 in colon cancer carcinogenesis and progression. For example, regression of small intestinal adenomas in ApcMin/+ mice induced by NSAIDs is blocked by PGE2 treatment [84]. Moreover, the adenoma-preventive activity of celecoxib is abrogated in 15-PGDH KO mice that possess increased endogenous PGE2 levels [85]. Genetic deletion of 15-PGDH increases endogenous PGE2 levels and promotes colon tumor growth also in ApcMin/+ and azoxymethane (AOM) mouse models [86]. In addition, intraperitoneal PGE2 treatment boosts the AOM-induced colon tumor incidence and multiplicity and significantly increases proliferative index and reduces apoptotic index [87]. Likewise, it has been demonstrated in three different mouse models of intestinal tumorigenesis that chronic low-dose aspirin prevents tumor formation and that the aspirin antitumor effect is most pronounced when treatment is started before tumor initiation [88].

To further outline the important role of PGE2 in colon carcinogenesis, different studies showed that mPGES-1 deletion suppresses the development of intestinal tumors in ApcMin/+ and AOM models [89,90]. Moreover, Sasaki et al. have reported that mPGES-1 deletion reduces AOM-induced colon polyp and aberrant crypt foci (ACF) formation [91]. Similar results were reported in animal models of breast cancer [92].

Additional information regarding the role of PGE2 signaling has been obtained from xenograft animal models, in which reduction of PGE2 production results in decreased tumor growth [15,26,93,94]. 

## 4. Molecular Mechanisms Linking PGE2 and Tumor Progression

**Figure 2:**
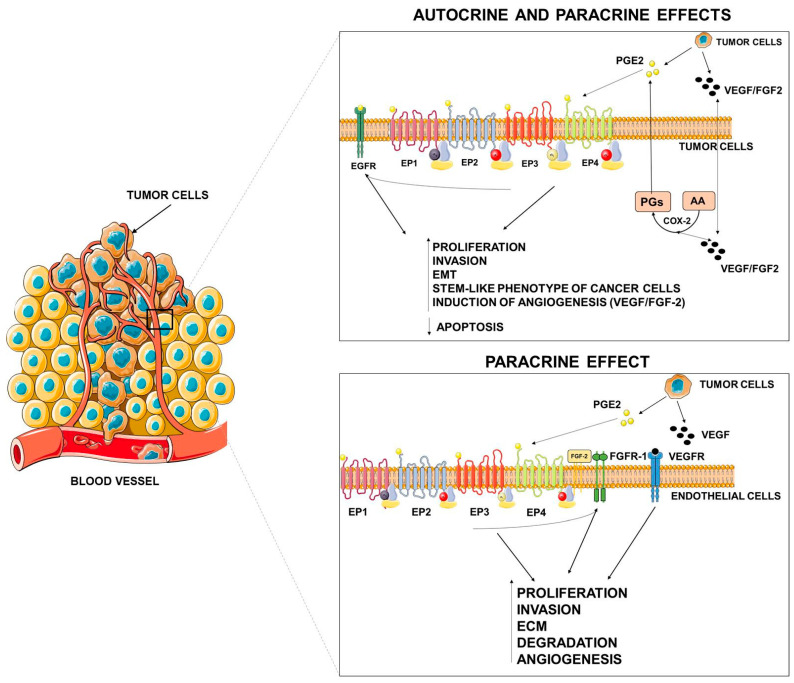
Autocrine and paracrine effects of PGE2. PGE2, released by tumor cells, may elicit autocrine and paracrine effects on tumor or stromal cells either by specific activation of its receptors or by tyrosine kinase (TK) receptor transactivation. VEGF = vascular endothelial growth factor, FGF = fibroblast growth factor, EMT = epithelial mesenchymal transition, ECM = extracellular matrix. This figure was created using Servier Medical Art templates, which are licensed under a Creative Commons Attribution 3.0 Unported License; https://smart.servier.com.

Inflammation has been reported as one of the hallmarks of cancer, due to its capability to supply bioactive molecules that promote cancer proliferation, invasion, and metastasis; limit cell apoptosis; and induce the angiogenic process [95]. Molecular and cellular inflammatory pathways that sustain cancer progression have been identified and are reported as intrinsic and extrinsic inflammatory pathways [96,97]. In the intrinsic pathway, genetic events that are able to induce neoplastic transformation promote the expression of inflammatory mediators that guide the construction of an inflammatory microenvironment and sustain tumor progression processes. Instead, the extrinsic pathway is driven by inflammatory leukocytes and soluble mediators that establish inflammatory conditions that increase cancer risk [96]. The upregulation of the COX-2/mPGES-1/PGE2 axis appears to be fundamental for both processes. In this review, we will provide a detailed overview of the principal molecular pathways activated by PGE2 (Figure 2).

As occurs in extrinsic inflammation, an increasingly large body of evidence indicates that PGE2 promotes tumor growth through autocrine and paracrine mechanisms by activating EP receptors present both in cancer cells and in stromal cells and by transactivating growth factor receptor tyrosine kinases (RTKs) frequently upregulated in cancer cells [15,98]. EP receptors activate a range of intracellular signaling pathways that mediate the effects of PGE2 on cell functions. 

The EP1 receptor is coupled to the Gαq protein subunit that is linked to phosphoinositide-PLC activation. This signaling leads to an increase of intracellular Ca2+ and PKC activation that finally induce gene transcription through the activation of nuclear factor of activated T cells (NFAT), nuclear factor-kappaB (NFκB), and the MAPK pathways [99]. 

The involvement of the EP1 receptor in cancer has been documented by several reports showing that EP1 signaling inhibition obtained by selective antagonists or by KO mice reduce the number of azoxymethane-induced aberrant crypt foci formation [100,101,102]. Consistently, EP1 receptor antagonists inhibit polyp formation in APC KO mice [103], decrease the number of UVB-induced skin tumors in mice [104], and diminish the incidence of tongue cancer in rats [105]. 

Instead, both the EP2 and EP4 receptors are linked to Gαs proteins that activate adenylate cyclase and generate cAMP which in turn activates the protein kinase A (PKA) pathway [106]. EP2 and EP4 receptors also mediate glycogen synthase kinase 3β (GSK3β)-β catenin pathways. PGE2 is able to promote colon cancer cell growth through EP2 signaling that involves the activation of phosphoinositide 3-kinase (PI3K) and the protein kinase Akt and the inactivation and release of GSK3β from its complex with axin, thereby activating the β-catenin signaling pathway [107]. Moreover, in similar models of colon cancer cells, PGE2 induces CREB phosphorylation by the PKA pathway and regulates β-catenin and cyclin D1 cellular localization via EP2 and EP4 receptors [108]. In addition, Akt pathway activation promoted by EP4 receptor results in the activation of mTORC1 [109]. Several other studies have shown that EP2 and EP4 induce the activation of multiple signaling cascades that are associated with squamous cell carcinoma (SCC) [110,111]; human hepatocellular carcinoma (HCC) [112]; glioma [113]; prostate [114], bladder [115], endometrial [116], and breast cancer [117] cell growth.

By using EP2 receptor KO mice, it has been demonstrated that the EP2 receptor controls the progression of lung [118], skin [119,120], and breast [121,122] cancer. Moreover, genetic ablation of the EP2 or EP4 receptors also decrease both the size and number of intestinal polyps in APC mice [123,124]. Moreover, the inhibition of the EP4 receptor with either AH23848 or ONO-AE3-208 reduces metastasis in breast cancer models [125]. 

The human EP3 gene consists of ten exons and nine introns, encoding at least eight distinct EP3 splice variants [126]. This could partially explain the different effects of EP3 in different tumors. The EP3 receptor is able to couple with a number of G-protein subunits including Gi, Gs, and G13, thus, stimulating or inhibiting adenylyl cyclase (AC), as well as stimulating Ca2+ mobilization, possibly via PLC. The major EP3 splice variant is thought to be coupled to an inhibitory (Gi) protein. Therefore, the primary outcome of EP3 receptor signaling is inhibition of AC and activation of the Ras/Raf and MAPK signaling pathway [106,127,128]. EP3 has been reported to mediate the carcinogenesis in numerous tumors with conflicting effects [15].

In addition to the canonical activation of EP receptors, PGE2 has been shown to promote cancer progression through the interaction with oncogenic signals, including epidermal growth factor (EGF) and its receptor (EGFR) [26,110,129,130,131,132]. In particular, PGE2 and EGF/EGFR may cooperate to promote growth, invasion, epithelial mesenchymal transition (EMT), and a stem-like phenotype of cancer cells [110,130,131,133]. It was also demonstrated that EGF induces the upregulation of mPGES-1 expression and PGE2 production, and that mPGES-1 inhibition significantly reduces the EGF-mediated tumorigenicity [26,93,134], indicating a cooperative loop between the two signaling pathways.

### PGE2 and Angiogenesis

Numerous in vitro and in vivo studies have indicated that the COX-2/mPGES-1/PGE2 pathway plays a pivotal role in promoting the angiogenic switch in cancer (Figure 2) [11]. COX-2/mPGES-1 overexpression in tumor cells has been reported to promote the production of angiogenic factors such as vascular endothelial growth factor (VEGF) and fibroblast growth factor (FGF-2) [11]. 

In cancer cells, PGE2 stimulates VEGF expression through several mechanisms including the activation of the hypoxia inducible factor-1α (HIF-1α) [135,136] or the cAMP signaling pathway [137]. Similarly, mPGES-1/PGE2 drive the angiogenic phenotype of cancer cells through the Dicer downregulation, and the subsequent PGE2-mediated downregulation of miR-15a and miR-186 that appear to be specifically related to VEGF production [138]. PGE2 can also induce VEGF secretion through the transactivation of EGFR mediated by EP2 and EP4 receptors [139,140,141]. Similarly, PGE2 can activate EP2-mediated FGF2 expression in endometrial adenocarcinoma cells through the activation of PKA, Src, EGFR, and ERK1/2 signaling [142].

Intriguingly, VEGF and FGF2 induce COX-2 expression and PGE2 production in endothelial cells. These data suggest that the effects of PGE2 on regulation of VEGF and FGF2 are probably amplified through a positive feedback loop [143,144].

In animal models, the EP2 receptor that controls the number and size of intestinal polyps in Apc (Delta 716) mice increases cellular cAMP and stimulates the expression of COX-2 and VEGF in the polyp stroma, demonstrating that PGE2 induction of VEGF is important for tumor growth in vivo [123]. Similar data have been reported also for breast cancer [122].

In addition to direct effects elicited by cancer-produced PGE2 on cancer and endothelial cells (autocrine/paracrine actions), several observations reported that PGE2 produced by stromal cells and present in the microenvironment may affect tumor niche to promote tumor progression. PGE2 signaling appears as a node of chronic inflammation which shapes the tumor microenvironment [145,146].

For example, PGE2 signaling in stromal cells also contributes to angiogenesis. A host PGE2–EP2 signal is required for tumor angiogenesis by enhancing endothelial cell motility and vascular hyperpermeability, as demonstrated in a mouse model of breast cancer in which the deletion of the EP2 receptor impairs tumor angiogenesis [147]. Similarly, the host PGE2–EP3 signal is a prerequisite for tumor-stromal angiogenesis that was markedly suppressed in mice EP3-/- and was linked to a reduced expression of VEGF [148].

PGE2 can also directly act on endothelial cells to promote a pro-angiogenic phenotype through the activation of fibroblast growth factor receptor-1 (FGFR1) signaling [149]. Indeed, it was reported that PGE2 synergizes with fibroblast growth factor 2 (FGF2) and induces the endothelial autocrine/paracrine FGF2/FGFR1 signaling through upregulation of FGF2 expression and its mobilization from the extracellular matrix [150]. In this context, PGE2 acts as a primer of the angiogenic switch by promoting the activation of the FGF2/FGFR1 system by multiple mechanisms [149,150]. 

Given the pivotal role played by the microenvironment in tumor metastasis, some observations are available on the possible molecular processes by which the primary tumor controls the pre-metastatic niche in the secondary site prior to the formation of metastasis. Liu et al. have determined that primary tumor-derived VEGF tumors can alter the lung microenvironment through PGE2 production in a model of breast cancer, leading circulating tumor cells to localize preferentially in these regions [151]. Similarly, PGE2 appears to regulate tumor metastasis in non-small-cell lung cancer (NSCLC) [152], colorectal cancer [153,154], breast cancer [155], and hypopharyngeal squamous cell carcinoma [156].

## 5. PGE2 and Its Roles in the Regulation of the Tumor Microenvironment 

**Figure 3:**
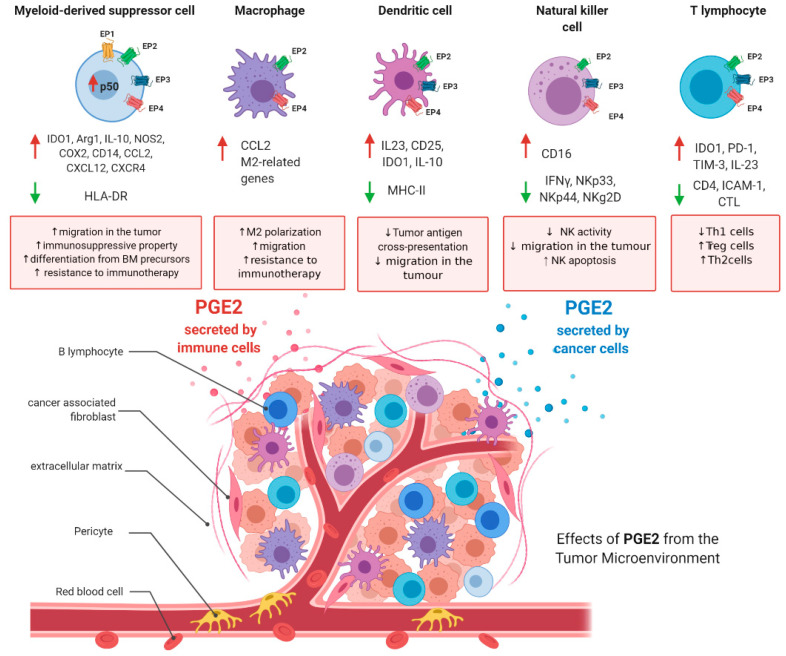
PGE2 roles in immunosuppressive tumoral niche. PGE2 levels are increased in the tumoral microenvironment. PGE2 is secreted by cancer cells and immune cells. In the figure are depicted the main functions of PGE2 in myeloid-derived suppressor cells, tumor-associated macrophages, dendritic cells, natural killer (NK) cells, and T-cells. Figure has been generated using BioRender.

It is now well accepted that a typical hallmark of tumors is an important immunosuppressive niche composed of suppressive immune cells which play a major role in the regulation of tumor progression, supporting cancer stemness and helping the tumor in the metastatic process. In the last decade, therapeutic strategies targeting these cellular populations have been developed and found to be beneficial in clinical practice (e.g., anti-PD1 and anti-CTLA4 therapies) [157]. The immune subsets that orchestrate tumor immunosuppression include myeloid-derived suppressor cells (MDSCs), tumor-associated macrophages (TAMs), dendritic cells (DCs), natural killer (NK) T cells, and regulatory T-cells (Tregs). Herein, as depicted in Figure 3, the major roles of PGE2 in the fine regulation of these cells are summarized, with the aim of immunoregulation. 

### 5.1. PGE2 and Myeloid-Derived Suppressor Cells 

Myeloid-derived suppressor cells (MDSCs) are a heterogeneous population of immature myeloid cells, enriched in cancers, with a crucial role in the maintenance of an immunosuppressive microenvironment [158]. Physiologically, in the bone marrow (BM), the hematopoietic stem cells (HSCs) give rise to immature myeloid cells (IMCs) which differentiate into mature myeloid cells. In the context of cancer, the tumor microenvironment releases mediators/cytokines that determine the development of IMCs to MDSCs. Furthermore, cancer cells secrete different types of chemokines that signal MDSC migration to tumors. MDSCs are schematically subdivided into monocytic-like cells (M-MDSCs) and granulocytic-like cells (PMN-MDSCs). These two subsets of MDSCs share the same ability to suppress adaptive immunity via different mechanisms of action. In brief, MDSCs suppress anti-tumor immunity through multiple mechanisms, including (i) the release of factors able to stimulate Treg activation and differentiation; (ii) the blockade of migration of naïve-T cells to lymphoid organs and the formation of effector T cells; (iii) production of high levels of reactive oxygen species (ROS) and nitric oxide (NO), up-regulating arginase 1 (ARG-1), and inducible nitric oxide synthase/ nucleotide-binding oligomerization domain-containing protein 2 (iNOS/NOD2). Of note, the induction of MDSCs’ development can be triggered by several factors including interleukin-1β (IL-1β), interleukin 10 (IL-10), macrophage-colony stimulating factor (M-CSF), interleukin-6 (IL-6), metabolic enzymes (e.g., NAMPT, indoleamine 2,3-dioxygenase (IDO1)), toll like receptor (TLR)-ligands, or VEGF [158,159,160]. Importantly, PGE2 has emerged as an important player in MDSCs’ activation and migration. 

#### 5.1.1. PGE2 and MDSCs Differentiation

The primary role of PGE2 in MDSCs was first characterized by Sinha and collaborators demonstrating how PGE2 controls MDSC differentiation in a preclinical model of mammary carcinoma. They highlighted that PGE2 induces a 3-fold increase in levels of suppressive Gr1^+^CD11b^+^ cells in vitro, therefore, prompting the idea that PGE2 is involved in the differentiation of MDSCs from bone marrow progenitors. Of note, by analyzing spleens of 4T1 tumor-bearing mice, they found that MDSCs express all the four receptors for PGE2 (i.e., EP1, EP2, EP3, EP4) [161]. Importantly, using butaprost (EP2 agonist), AH6809 (EP1 and EP2 antagonist), and AH23848 (EP4 antagonist), they clearly demonstrated that PGE2 mediates MDSC differentiation through either the EP1, EP2, and/or EP4 receptors. Using EP2 KO mice they also proved that the ablation of EP2 receptor retards mammary carcinoma growth by reducing MDSC levels, indicating that PGE2 mediates MDSC accumulation also in vivo [161]. Importantly, the treatment with the COX-2 inhibitors (SC58236 and SC58236) delays primary carcinoma tumor burden with an important reduction of MDSCs levels. 

In accordance with these findings, it is known that tumor exosomes are taken up by bone marrow myeloid cells and contribute to the development of MDSCs [162]. Furthermore, Xiang et al. demonstrated that the promotion of tumor growth by MDSCs is dependent on tumoral exosomes which are enriched of PGE2. Of note, antibodies against exosomal PGE2 block the activity of these exosomes on MDSCs’ induction and attenuate tumor growth in vivo [163]. In agreement with this study, the direct role of PGE2 in the activation of monocyte differentiation has been proven since the stimulation of monocytes with PGE2 induces several immunosuppressive factors leading to MDSC phenotype (e.g., IDO1, Arg1, IL-10, NOS2). Moreover, PGE2 per se induces COX-2 leading to autocrine production of endogenous PGE2 in MDSCs [164]. Obermayer and collaborators discovered a positive feedback loop between PGE2 and COX-2 in the differentiation of monocytes, redirecting to functional differentiation of DCs toward monocytic MDSCs, a mechanism undertaken by cancer cells to locally produce suppressive MDSCs. Alone, the administration in vitro of PGE2 redirects the granulocyte-macrophage colony-stimulating factor (GM-CSF) and IL-4 to the differentiation of dendritic cells toward MDSCs [164,165]. The ability of EP2 and EP4 agonists, but not of EP3/1 agonists, to reproduce PGE2-induced effects demonstrates the key role of EP2 and EP4 in mediating the MDSCs. Of note, Rodriguez-Ubreva et al. studied the comparison between MDSC and DC DNA methylomes and revealed extensive demethylation with specific gains of DNA methylation. In particular, they found that PGE2 leads to highly specific and DNA methyltransferase 3 alpha (DNMT3A)-dependent hypermethylation and downregulation of a subset of myeloid-associated genes. Although the mechanisms by which MDSCs suppress T-cells have been extensively studied, less is known about MDSCs’ regulation of NK-cell activity [166]. Direct involvement of PGE2 during the initiation and maintenance of NK-cell suppression by MDSCs has been proven by Mao et al. They demonstrated that PGE2 increases the immature status of monocytes. Indeed, PGE2 increases the expression of CD14, while the expression of HLA-DR is reduced [167]. They found that the co-culture of MDSCs isolated from melanoma patients with NK cells induces interferon (IFN)γ release and IL2-activated NK cells. The stimulation with PGE2 suppresses NK-cell responses, TGFβ production, and the activation of the EP2 or EP4 receptors [167].

#### 5.1.2. PGE2 and MDSCs Recruitment

Several reports have suggested a key role of PGE2 in the recruitment of MDSCs in the tumor site. It is well known that the CXCR4-CXCL12 axis is the key pathway involved in the recruitment of MDSCs into the tumor. Obermajer et al. have observed a correlation between the expression of COX-2, CXCL12 concentrations, and the production of PGE2 in ovarian cancer. Importantly, the secretion of CXCL12 in ovarian cancer ascites was hindered by COX-2 inhibitors [164]. They also found that PGE2 induces both CXCL12 production in the ovarian cancer environment and CXCR4 expression in MDSC precursors, therefore, promoting the attraction and retention of MDSCs in the tumor microenvironment [164]. Similarly, in mouse models of glioma, it has been demonstrated that treatment with COX-2 inhibitors reduces the PGE2 production which results in a decrease of MDSC-attracting chemokine CCL2 in the tumor. These data suggest that COX-2 blockade hampers the development of MDSCs and their accumulation in the tumor with a CCL2-dependent mechanism [168]. The role of COX-2 and PGE2 in the recruitment of MDSCs has been validated also in lung cancer. The authors demonstrated that cytotoxic T lymphocytes (CTLs) induce tumor cells to secrete PGE2 as a mediator of MDSCs’ recruitment into the tumor. Indeed, both the COX-2 inhibitor and the PGE2 neutralizing antibody (2B5) reduce the number of MDSCs recruited by tumor cells [169]. All these data suggest that the chemoattraction of MDSCs by tumor cells is mediated first by the up-regulation of COX-2 and, secondly, by the PGE2 synthesis. 

#### 5.1.3. PGE2 and MDSCs Activation

Within the tumors, MDSCs are activated and are able to suppress T-cell proliferation, resulting in the impairment of the anti-tumor immunity. Activated MDSCs increase the expression of iNOS/NOD2, arginase, and IDO1 leading to a significant increase of ROS and NO levels and finally to a decrease of T-cells’ proliferation [170]. 

Ochoa et al. have described for the first time that PGE2 secreted into the tumor microenvironment is a signal to increase arginase expression in MDSCs, resulting in the block of T-cells’ proliferation [171]. In addition, the Kiessling group found that the axis COX-2/PGE2 is at the basis of the mechanism by which melanoma cells arrest monocytes in an immature stage, similar to the stage of tumor MDSCs, associated with the ability to impair T-cell functions. Indeed, they found that treatment of melanoma-patient-derived monocytes with PGE2 leads to the loss of T-cell proliferation and IFNγ production. Of note, the suppression is mediated by the direct activation of monocytes despite T cells, because PGE2 per se does not affect T-cell proliferation in this cellular model [172]. 

Recently, Porta et al. clearly demonstrated that tumor-derived PGE2 leads to the nuclear accumulation of p50-NF-κB in M-MDSCs, resulting in a reduction of tumor necrosis factor (TNF)α expression and diverting their response to IFNγ. In agreement, the treatment with butaprost (PGE2 receptor antagonist) is able to reprogram M-MDSCs towards a “NOS2^low^/TNFα^high^ phenotype”, thus, restoring the antitumor activity of IFNγ [160]. An important role of MDSCs in the tumor is also to control cancer stem-like cells. It has been demonstrated both in vitro and in vivo that MDSCs induced by tumor-derived G-CSF enhance the stemness of cervical cancer cells by producing PGE2. Moreover, PGE2 produced by MDSC increases tumor PD-L1 expression in ovarian cancer cells, in an mTOR-dependent mechanism. Accordingly, the treatment with celecoxib inhibits the induction of cancer stem-like cells and enhances the efficacy of common chemotherapy (e.g. cisplatin). Importantly, the authors have translated these pieces of evidence for humans and they found a positive correlation between MDSCs, PGE2, and CSCs in clinical samples [173,174]. 

#### 5.1.4. PGE2, MDSCs, and Resistance to Immunotherapy

Although immunotherapies have been found effective in several types of cancers (e.g., anti–PD-L1 and anti-PD-1 therapies), a portion of patients still fail to respond to therapy, and there is an urgent need to discover the mechanism at the basis of this resistance. In this setting, Prima et al. found that co-culture of BM cells with bladder tumor cells promotes strong expression of PD-L1 in M-MDSCs [161]. Tumor-infiltrating PD-L1^+^ cells isolated from tumor-bearing mice express high levels of PGE2 synthase 1 (mPGES1) and COX-2. Interestingly, the treatment with mPGES1/COX-2 inhibitors reduces the PD-L1 expression in MDSCs, suggesting that reprogramming PGE2 metabolism in a tumor microenvironment provides an opportunity to reduce immune suppression and may increase the efficacy of immunotherapy. Moreover, Hou et al. demonstrated that tumoral PGE2 is a key mediator of immunotherapy resistance (including virotherapy). Indeed, they have highlighted a decrease of Treg, but not of the number of MDSCs, after viral therapy, in different tumor models (e.g., 4T1, MC38), suggesting that MDSCs can block the immunotherapeutic activity of these vectors. The authors found a correlation between elevated levels of PGE2 and the suppressive profiles of tumors as well as with high levels of PMN-MDSCs, and using viral vectors engineered to target PGE2, they were able to alter the cancer immune status [175]. 

### 5.2. PGE2 and Tumor-Associated Macrophages

Macrophages display marked plasticity since they can polarize to M1- (pro-inflammatory) and M2-like (anti-inflammatory) phenotypes by various stimulations (e.g., IFNγ, LPS, IL-4) in inflamed tissues or in cancers. In a tumoral setting, it is well accepted that macrophages acquire the M2-like phenotype and become the so-called tumor-associated macrophages (TAMs). They could promote migration, metastatization, and angiogenesis and suppress regulatory immune circuits.

#### 5.2.1. Role of PGE2 in Controlling Macrophage Polarization

The key role of PGE2 in the regulation of macrophage polarization has been extensively described. Indeed, it has been demonstrated that human peripheral blood mononuclear cells (PBMCs) cultured in the presence of GM-CSF and IL-4 normally differentiate into DCs, while the stimulation with PGE2 suppresses the formation of DCs and shifts the differentiation into the M2-like macrophages. The mechanism at the basis of this switch in the differentiation process seems to be dependent on the activation of EP4, since the treatment with E7046 (EP4 antagonist) is able to revert the differentiation to M2-like macrophages [176,177,178,179]. These data prove that PGE2 promotes M2 polarization, therefore, contributing to the enhancement of the anti-tumor immunity. In accordance, macrophages usually express EP2 and EP4, but not EP1 or EP3. The treatment with EP4 antagonist (E7046) of ApcMin/+ mice determines the change of TAM phenotype from M2 to M1 polarization, suggesting that EP4 is essential for PGE2-dependent M2 polarization [177]. Similarly, it has been proved in a glioblastoma model that PGE2, secreted by glioblastoma cancer stem cells, is able to convert macrophages into M2-TAMs [176]. Finally, as already reported in the MDSC section, the activation of COX-2/mPGES1/PGE2 pathways promotes PD-L1 expression by TAMs [180].

#### 5.2.2. Role of PGE2 in Controlling Macrophage Migration

PGE2 controls also macrophage migration into the tumor. Indeed, it has been demonstrated that PGE2 leads to CCL2 up-regulation, a key chemokine involved in macrophage recruitment in the tumor. Moreover, in a mouse model of gastric cancer, the treatment with EP4 antagonist reduces the recruitment of M2-like macrophages and consequently the tumoral growth [179]. 

### 5.3. PGE2 and Dendritic Cells 

Dendritic cells (DCs) are able to exert different activities including being sentinels of the immune system, checking continuously the immune niche. DCs are not effector cells against pathogens, but they control adaptive immunity, presenting the foreign antigens [181]. All DCs originate from a macrophage/dendritic cell progenitor (MDP) present in the bone marrow which further differentiates itself into the monocyte/macrophage lineage or to the common dendritic cell progenitor (CDP). CDP in the bone marrow differentiates in turn to both plasmacytoid DC (pDC) and pre-DC progenitors. pDCs go to the bloodstream as mature functional cells. Therefore, pre-DCs migrate through the vascular system to their final location in the tissues or lymph nodes, where they differentiate into conventional DCs (cDCs) [182,183]. Apart from antigen presentation, DCs deliver co-stimulatory signals and produce cytokines which are necessary for instructing appropriate effector or regulatory T-cell responses. Antigen-presenting cells, as dendritic cells, are able to guide immune response to tumor antigens. Circulating DC levels and activity are reduced in cancer patients as compared with healthy subjects, correlating with the severity of disease. 

#### 5.3.1. PGE2 and DC Differentiation

Notably, a key role of PGE in controlling DC differentiation has been highlighted by different authors. Indeed, in tumor models of colon cancers, PGE2 promotes tumor growth by suppressing DC differentiation from bone marrow progenitors [79]. Furthermore, PGE2 inhibits the antigen presentation ability of BM-derived DCs by reduction of MHC II expression and upregulation of IL-10 through EP2 and EP4 [79]. PGE2 has also been shown to switch the function of DCs from induction of immunity to T-cell tolerance via upregulation of CD25 and indoleamine 2,3-dioxygenase (IDO1). Furthermore, PGE2 unbalances the IL-12/IL-23 axis using EP2 and EP4 receptors in favor of IL-23 which brings an increase of the number of Th17 cells in vitro [79]. 

Importantly, Ogawa et al. have reported that COX-2-derived PGE2 is essential for the formation of a premetastatic niche and lymph node metastasis (LNM). Using a murine model of Lewis lung carcinoma (LLC), they have shown COX-2 overexpression in cDCs. The administration of a COX-2 inhibitor, plus a stromal cell-derived factor 1 (SDF-1) antagonist and a CXCR4 neutralizing antibody, determine the reduction of LNM. Moreover, LNM is reduced in KO mice for EP3, suggesting that the effect of PGE2 on DCs could be mediated by the activation of EP3, despite other receptors. Indeed, compared with WT CD11c^+^ DCs, injection of EP3-deficient CD11c^+^ DCs dramatically reduces accumulation of SDF-1^+^CD11c^+^ DCs in regional lymph nodes (LNs) and lymph nodes metastasis (LNM) in LLC-injected mice, showing a COX-2/EP3-dependent signaling [184]. 

#### 5.3.2. PGE2 and DC Migration

Migration and storing of cDCs to lymphoid organs are essential for T-cell-induced response against tumors. However, tumor niche might let some tumor cells escape from the immune response by reducing DC migration. A key role of PGE2 in this setting has been proven. The medium of murine prostate cancer cells inhibits migration of BM-DCs and splenic cDCs through the activation of CC chemokine receptor-7 (CCR7) ligand CCL19 in vitro, and migration to draining lymph nodes in vivo [185]. However, the treatment with PGE2 rescues this impairment of DC migration with upregulation of CCR7 and inhibition of LXRα. Moreover, in prostate-cancer-bearing mice, PGE2 treatment inhibits tumor growth and induces more tumor-infiltrating T cells and CD11c dendritic cells in tumor niche [185].

### 5.4. PGE2 and Natural Killer Cells

Natural killer (NK) cells are innate immune lymphocytes, with a role in the regulation of innate and adaptive immune response which primarily function to lyse tumor cells. NK cells targeting tumor-mediated mechanisms include granule exocytosis, death receptor-mediated killing, and interferon (IFN)-γ release [186]. Activation receptors are NK group 2, member D (NKG2D) and natural cytotoxicity receptors NKp44, NKp46, and NKp30. NK cells produce perforin and granzyme B to penetrate into target cells and bring them to death. Activated NK cells also secrete IFN-γ to stimulate other immune cell types and activate an immune response [186]. Various types of tumor cells express ligands that are recognized by NK cells and stimulate their cytotoxic activity. NKG2D binds major histocompatibility complex class I polypeptide-related sequence A/B expressed on the surface of cancer cells, proliferating cell nuclear antigen binds to NKp44, while B7-H6 molecule is recognized by NKp30. Usually, ligands are more expressed in tumor cells than in normal cells [187]. 

#### PGE2 and NK Activity

The general idea is that NK cells could reject human tumors, influencing clinical outcome. In 1992, Fulton et al. have firstly evaluated that PGE2 receptor, without distinguishing the isoform, is overexpressed in metastatic murine mammary tumor, and PGE2 itself is able to induce NK activity inhibition [188]. Of note, NK cells express EP2, EP3, and EP4, but not EP1 [189]. Several manuscripts, and several years later, it has been highlighted the efficacy of NK to target cancer cells, whose activity is punctually suppressed by PGE2 production, usually secreted by tumoral cells. Suppressed NK cell activity has been found in human colorectal cancer and it is an important prognostic factor for the development of metastases [79]. Similarly, tumor-infiltrating NK cell levels are associated with an improved rate of survival in gastric cancer, negatively correlating with COX-2 levels, and enhancing lung metastases in rats [79]. Moreover, modulation of EP4 receptor signaling mediates the effects of PGE2 on the promotion of breast cancer metastasis and suppression of NK cell function in a murine model of metastatic breast cancer, suggesting that EP4 may be crucial for the activation of NK by PGE2. In this case, PGE2 suppresses NK cell function via multiple mechanisms: (i) downregulating NK receptors via a cAMP/PKA pathway, (ii) inhibiting production of IFN-γ by NK cells and IL-12–induced or IL-18–induced IFN-γ expression in NK cells via EP2 receptor, and (iii) inhibiting NK cell proliferation and inducing apoptosis [79]. PGE2 inhibits the killing of target cells by NK cells activated through NCR, CD16, or NKG2D. Moreover, the percentage of CD107a^+^ NK cells is significantly inhibited by increasing doses of PGE2 in a dose-dependent manner [189]. Both melanoma and hepatocellular carcinoma cells are able to inhibit the expression of NK receptors that trigger their immune function, including NKp30, NKp44, and NKG2D, with the impairment of NK-cell-mediated cytolytic activity against melanoma cells, through IDO1 and PGE2 expression [190,191]. Moreover, Park et al. have also highlighted that thyroid cancer cells suppress the cytolytic activity of NK cells through PGE2 secretion, downregulating NKp44 and NKp30 receptors. They have described that PGE2 and COX-2 are over-expressed in anaplastic thyroid cancer cells [192] and that also cancer-associated fibroblasts (CAFs) are responsible for PGE2 production [193]. Several NK functions, such as lysis, migration, and cytokine production, are compromised in tumor-bearing (injected with 66.1 cells) mice, producing PGE2. Indeed, PGE2 interferes with the potential of NK cells to migrate, exerting cytotoxic activity, and secreting IFNγ, with a mechanism dependent on the activation of EP2 and EP4 receptors. Importantly, NK cells are susceptible to inhibition after the treatment with EP4 and EP2 agonists compared to NK cells from healthy mice. Holt et al. have reported that an EP4 antagonist (frondoside A) inhibits breast tumor metastasis in an NK-dependent manner and protects IFNγ production by NK cells from PGE2 mediated suppression [194,195]. Similarly, the EP4 antagonist AH23848 reduces the ability of tumor cells to colonize the lungs or to spontaneously metastasize from the mammary gland. Of note, metastasis inhibition is lost in mice lacking either functional NK cells or interferon-γ [196]. Lastly, unique molecule EP4 antagonist (RQ-15986) is able to reduce tumoral mass in a syngeneic murine model of metastatic breast cancer. NK-cell functions are markedly depressed in mice bearing murine mammary tumor 66.1 or 410.4 cells due to the actions of PGE2 on NK cell EP4 receptors [197]. Taken together, all these reports clearly demonstrate that the activation of the EP4 receptor is essential for the PGE2 activation of NK cells. 

Another emerging role of NKs is that they are able to crosstalk with both T cells and dendritic cells. The cytokine- and chemokine-producing capacity, T-cell polarization, migration, and stimulatory functions of DCs are finely regulated by activated NK cells. NK functions require close interactions with activated DCs. Cell-membrane-associated molecules and soluble mediators, including cytokines and prostaglandins, contribute to the bidirectional crosstalk between DCs and NK cells, usually inhibited by PGE2 [198].

### 5.5. PGE2 and T-Cells 

HSCs differentiate into multipotent progenitors (MPPs) which could be differentiated in both myeloid and lymphoid cells. MPPs differentiate to a common lymphoid progenitor (CLP) which converts exclusively into T, B, or NK cells. CLPs migrate to the thymus, where they implant themselves. First, cells that implant in the thymus are the double-negative ones, as they express neither the CD4 nor the CD8 co-receptor, while double positive CD4/CD8 migrate into the cortex of the thymus and present self-antigens. If they interact with MHC-I or MHC-II, they survive; if they do not, they will be discarded. If the double-positive cells interact with MHC class II, molecules will differentiate in CD4^+^ cells—CD8^+^ cells if they interact with MHC class I [199]. Differentiated T cells have an important role in adaptive immunity and are subdivided into (i) CD8^+^ T cells that have cytotoxic activity, able to kill infected or tumor cells; (ii) CD4^+^ T cells, or "helper cells”, that release cytokines and activate indirectly regulatory B cells; and (iii) regulatory T cells (Treg) usually activated by tumor cells to prevent their killing. 

Several studies have underlined the effect of PGE2 on T cells. For example, it has been reported that PGE2 is able to inhibit the proliferation of T cells in a dose-dependent manner in vitro. A comparison between IFN-γ and IL-4 production showed that PGE2 increases the relative ratio of IL-4 to IFN-γ in CD4^+^ T cell culture and regulates CD4^+^ T cells toward Th2 development. This mechanism seems to be primarily dependent on IDO-overexpression, determining Tregs formation and development [200]. In support of these findings, it has been reported that murine renal carcinoma (Renca) cells over-secrete PGE2 and consequently inhibit antitumor cytotoxic T lymphocyte (CTL) responses in vivo by preventing the IFNγ-dependent upregulation of ICAM-1 responsible for the initial priming of naïve CD8^+^ T cells. In addition, exogenous IFNγ abolishes PGE2-mediated suppression on naïve CD8 T-cell priming, and overexpression of ICAM-1 by tumor cells re-establishes IFNγ production [201]. PGE2 immunosuppression may be an indirect consequence determined by COX-2 overexpression, as in melanoma. In a number of tumor cell lines, constitutive IDO1 expression depends on COX-2 and PGE2 which upon autocrine signaling through the EP receptor activates IDO1 via the PKC and PI3K pathways. Moreover, most of these tumors have been associated with PI3K or MAPK mutations which may support constitutive IDO1 expression, and usually lack T-cell infiltration and fail to immunotherapy [202].

CD4^+^CD25^+^ T reg cells play an important role in the maintenance of immunologic self-tolerance in non-small cell lung cancer. CD4^+^CD25^+^ T reg cell activities increase in lung cancer and appear to play an important role in suppressing antitumor immune responses. COX-2/PGE2 signaling induces expression of Foxp3 and increases Treg properties. PGE2-mediated induction of Treg cell Foxp3 gene expression is significantly down-expressed in the absence of the EP4 receptor and totally eliminated in the absence of the EP2 receptor. In vivo, COX-2 inhibition reduces Treg cell frequency and activity, attenuates Foxp3 expression in tumor-infiltrating lymphocytes (TILs), and decreases tumor weight. Adoptive Treg cells transfer or administration of PGE2 to mice treated with COX-2 inhibitor are able to overturn these effects [203]. Another important piece of evidence is reported by Kim et al. who have emphasized GM-CSF potential in cancer vaccines through IL9-producing Th (Th9) cells. GM-CSF improves Th9 cell differentiation by regulating the COX-2–PGE2 pathway and it is able to inhibit the differentiation of induced regulatory T cells in vitro and in vivo. GM-CSF-activated monocyte-derived dendritic cells convert tumor-specific naïve Th cells into Th9 cells and delay tumor growth by inducing antitumor CTLs in an IL9-dependent manner [204]. 

Moreover, fibroblastic reticular cells (FRCs) in the T-cell zone of lymph nodes are essential for T-cell survival, mobility, and tolerance. FRCs are able to limit T-cell activation, secreting PGE2 due to COX-2 overexpression in immune cells [205]. Another important role of PGE2, mainly related to immunotherapy resistance, is its ability to promote the expression of PD-1 and TIM-3 in T-cells increasing the interaction with PD-L1 and PD-L2, highly expressed on CAFs. Blocking the activity of PGE2 partially restores the proliferative capacity of both CD4^+^ and CD8^+^ T-cells [206].

All these reports demonstrate an important function of PGE2 in controlling the balance between Th1, Th2, and Treg, with a key role in the induction of Treg and Th2 phenotype resulting in a promotion of immunosuppressive niche and tumor growth.

## 6. Conclusions

The importance of inflammation in driving the predisposition to cancer has been widely documented. Starting from this observation, in recent years the scientific community has begun to repurpose anti-inflammatory agents not only for the prevention but also as a therapeutic option in cancer. Among the inflammatory mediators, PGE2 is known to play a pivotal role in regulating the inflammatory milieu that drives cancer onset and progression. Several studies have demonstrated that PGE2 is able to activate growth factor signaling; promote cancer cell growth and resistance to apoptosis and metastasis; and to modulate immune response. Extensive clinical and epidemiological studies support the idea that PGE2 level reduction could be useful to prevent tumor initiation, to reprogram anti-tumor immunity, to inhibit tumor growth and metastasis, and, finally, to increase the efficacy of current pharmacological and immunological therapies. 

To date, the anti-tumoral potential of aspirin and coxibs has been well studied in preclinical and clinical settings. Notably, several clinical trials aimed to study the efficacy of coxibs alone or in combination are under investigation. For example, the clinical efficacy of celecoxib alone is under phase I and II clinical trials for treating patients with stage I, stage II, or stage IIIA non-small cell lung cancer and advanced carcinoma of the cervix (NCT00030407 and NCT00023660 clinicaltrials.gov). Further, the combination of celecoxib with irinotecan, cisplatin, and radiation therapy is under investigation in esophageal cancer (NCT00023660 clinicaltrials.gov). 

Of note, the current therapies targeting PGE2 using NSAIDs or COX-2 inhibitors have sometimes failed due to the global prostanoid suppression which in turn results in severe side effects [207]. It is, therefore, more plausible and clinically relevant to act not on PGE2 biosynthesis, but mainly on the antagonism of EP receptors. For this reason, numerous small-molecule ligands targeting EP receptors have been developed and are under investigation both as conventional anticancer agents and as immunomodulating drugs (e.g., ONO-8711 for EP1; PF-04418948 for EP2; ONO-AE3-240 for EP3; AH23848b for EP4). For example, the antagonist of EP4, RQ-00000007, is under clinical evaluation in combination with gemcitabine for prostate cancer, non-small cell lung cancer, and breast cancer (NCT02538432 www.clinicaltrials.gov). The TPST-1495 dual EP2 and EP4 antagonist is in phase 1a/1b as single treatment or in association with pembrolizumab in solid tumors (bladder cancer, triple negative breast cancer, gastric cancer) (NCT04344795 clinicaltrials.gov).

This review has highlighted the pleiotropic role of PGE2 in controlling tumor cells but also the tumor microenvironment (TME), suggesting that targeting PGE2 could be a good strategy to both act on cancer cells but also on immune cells. Accordingly, the putative immunomodulatory effect of blocking the PGE2 pathway is under investigation. For example, a phase I clinical study has already been started with E7046, an EP4 inhibitor, with a potent immunosuppressive effect on myeloid cells in the TME (NCT02540291: https://clinicaltrials.gov/ct2/show/study/NCT02540291).

In conclusion, PGE2 represents an old target with pleiotropic functions in TME with a new potential clinical impact. The usage of NSAIDS and COX-2-inhibitors as anti-cancer agents may be bypassed by selective EP antagonists which may overcome severe side effects and, therefore, increase the real potential of targeting the PGE2 pathway as a novel therapeutic approach in the clinical setting.

## Figures and Tables

**Table 1:** Studies related to the role of aspirin and other non-steroidal anti-inflammatory drugs (NSAIDs) in cancer.

NSAID	Cancer	References
Aspirin	Colorectal cancer	[3,18,32,33,34,35,36,37,38,39,40,41,42,43,44,45]
	Breast cancer	[17,41,46,47,48,49,50,51]
	Head and neck squamous cell carcinoma	[52,53,54,55,56,57,58,59,60]
	Lung cancer	[3,37,38,43,61]
	Prostate cancer	[3,62]
	Ovarian cancer	[37,63,64,65]
	Gastric cancer	[3,37,66,67,68,69,70]
Coxib	Breast cancer	[31,71,72,73,74,75,76]
	Colon cancer	[31,76,77,78,79]
	Prostate cancer	[31,76]
	Lung cancer	[31,76,80,81,82,83]
